# Direct RNA Sequencing of Foot-and-mouth Disease Virus Genome Using a Flongle on MinION

**DOI:** 10.21769/BioProtoc.5017

**Published:** 2024-06-20

**Authors:** Lizhe Xu, Amy Berninger, Steven M. Lakin, Vivian O’Donnell, Jim L. Pierce, Steven J. Pauszek, Roger W. Barrette, Bonto Faburay

**Affiliations:** 1 Foreign Animal Disease Diagnostic Laboratory (FADDL), National Veterinary Services Laboratories, Animal and Plant Health Inspection Service, U.S. Department of Agriculture, Plum Island Animal Disease Center, Plum Island, New York, NY, USA; 2 Oak Ridge Institute for Science and Education (ORISE), Oak Ridge, TN, USA; 3 FADDL, National Bio- and Agro-defense Facility (NBAF), United States Department of Agriculture, Manhattan, KS, USA

**Keywords:** Foot-and-mouth disease virus, FMDV, Nanopore sequencing, Direct RNA Sequencing, NGS, Point of care

## Abstract

Foot-and-mouth disease (FMD) is a severe and extremely contagious viral disease of cloven-hoofed domestic and wild animals, which leads to serious economic losses to the livestock industry globally. FMD is caused by the FMD virus (FMDV), a positive-strand RNA virus that belongs to the genus *Aphthovirus*, within the family *Picornaviridae*. Early detection and characterization of FMDV strains are key factors to control new outbreaks and prevent the spread of the disease. Here, we describe a direct RNA sequencing method using Oxford Nanopore Technology (ONT) Flongle flow cells on MinION Mk1C (or GridION) to characterize FMDV. This is a rapid, low cost, and easily deployed point of care (POC) method for a near real-time characterization of FMDV in endemic areas or outbreak investigation sites.

Key features

Saves ~35 min of the original protocol time by omitting the reverse transcription step and lowers the costs of reagents and consumables.

Replaces the GridION flow cell from the original protocol with the Flongle, which saves ~90% on the flow cell cost.

Combines the NGS benchwork with a modified version of our African swine fever virus (ASFV) fast analysis pipeline to achieve FMDV characterization within minutes.

Graphical overview

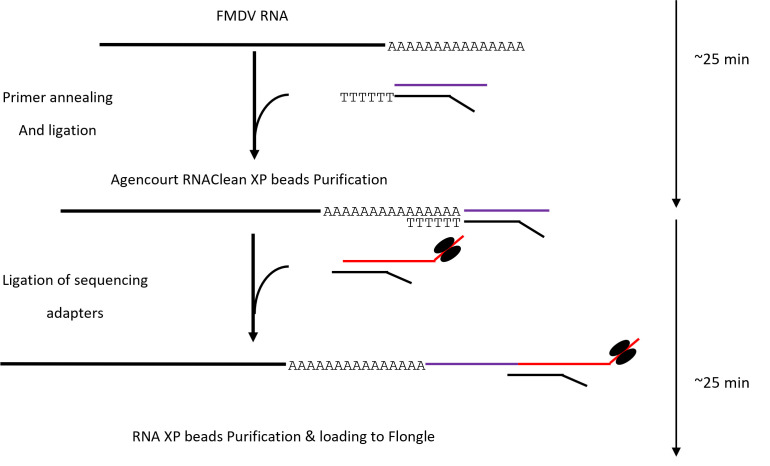

**Schematic of direct RNA sequencing of foot-and-mouth disease virus (FMDV) process, which takes ~50 min from extracted RNA to final loading, modified from the ONT SQK-RNA002 protocol (Version: DRS_9080_v2_revO_14Aug2019).**

## Background

Foot-and-mouth disease (FMD) is a severe and extremely contagious viral disease that causes vesicular disease in cloven-hoofed domestic and wild animals. Outbreaks can lead to serious economic losses due to decreased livestock productivity and restrictions on movement and trade of animals and their products [1]. The causative agent of the disease is foot-and-mouth disease virus (FMDV), an *Aphthovirus* from the family Picornaviridae, with seven antigenically distinct serotypes including the EuroAsiatic serotypes A, O, C, and Asia 1, and the Southern African territories (SAT) serotypes 1, 2, and 3. Cross-protection is not conferred between serotypes following infection or vaccination and may not be conferred between different subtypes or variants of the same serotype [2]. The key factor to prevent the disease and control its spread is early detection and identification of the serotype(s) and subtype(s) of outbreak strains so that directed vaccination may be initiated. The antigenic determinants of FMDV reside within the structural proteins of the viral capsid (VP1-VP4), encoded by the genes 1A (VP4), 1B (VP2), 1C (VP3), and 1D (VP1). Historically, FMDV molecular epidemiology of outbreaks has focused on analyses of partial or full-length sequences of the 1D gene that encodes the structural protein VP1 [3]. Currently, over 60% (9571/14952 in December 2023) of FMDV records in the GenBank database have a total nucleic acid length of 400–700 bp and cover the partial or full 1D gene. Unfortunately, relying solely on the VP1 coding sequence does not provide all of the information required to fully characterize important phenotypic traits of FMDV strains, since several antigenic determinants are located on other viral capsid proteins, such as VP2 and VP3 [4,5]. Moreover, during shorter epidemic time scales, sequencing of 1D cannot provide enough resolution to discriminate the viruses collected from adjacent premises within the same outbreak clusters due to the viral populations not having diverged substantially [6]. In this regard, numerous reports have been published using the whole P1 region, encoding all structural proteins (i.e., VP1 to VP4) for strain genotyping and phylogenetic analysis [7,8]. The characterization of the whole P1 region (1A to 1D) is important for tracking the emergence or spread of FMD and for selecting vaccines in case of an outbreak. However, restricting analyses to P1 sequences may not detect recombination events that can occur between multiple serotypes or topotypes in the event of co-circulating FMDV within infected animals [5,9,10]. Next-generation sequencing (NGS) techniques offer much promise to perform viral whole genome sequencing (WGS) without prior knowledge about the target sequence and provide a huge amount of data from a limited quantity of starting material in a rapid, cost-effective manner. Valdazo-González and colleagues (2011) have demonstrated that WGS of FMDV is a powerful tool for the reconstruction of transmission trees.

WGS of FMDV has been previously conducted on different platforms including Oxford Nanopore Technology (ONT) and Illumina [6,10,12–13]. These methods use reverse transcriptase to convert RNA to cDNA and/or go through one or two FMDV-specific or non-specific PCR amplifications. Some are labor-intensive with cumbersome procedures; others may have relatively longer turnaround times. Moreover, viral RNA manipulation can introduce biases into the data [14], which may not represent the actual diversity within samples [6]. Here, we introduce for the first time an FMDV direct RNA sequencing method using Nanopore sequencing on Flongle flow cells with MinION Mk1C (or GridION). Compared to the original ONT SQK-RNA002 protocol, we omitted the optional reverse transcription step to make the process even shorter by saving ~35 minutes as the ONT device only sequences the RNA molecule, not the cDNA. Our method is low-cost, fast, and reliable and can be deployed to low-resource settings when using an Mk1C device, such as a remote or field laboratory and an alternative RNA extraction method. This method requires less than 50 min of manipulation time from RNA to NGS run (Graphical Abstract), followed by a rapid identification of the whole FMDV genome within minutes.

## Materials and reagents

**Biological materials**

Seven FMDV isolates were used to develop the RNA direct sequencing method, one of each serotype (see Table 4 in the Evaluation of Protocol section below). The isolates were obtained from the Biorepository in the Reagents and Vaccine Services Section of FADDL as viral stocks propagated from cell lines identified in Table 4.

**Reagents**

1. MagMAX^TM^ CORE Nucleic Acid Purification kit (Thermo Fisher, catalog number: A32702 or A32700)
2. Qubit RNA High Sensitivity (HS) Assay kit (Thermo Fisher, catalog number: Q32852 or Q32855)
3. Ethyl alcohol, molecular grade (e.g., Thermo Fisher, catalog number: T032021000)
4. RNAClean XP beads (Beckman Coulter, catalog number: A63987)
5. Direct RNA Sequencing kit (ONT, catalog number: SQK-RNA002)
6. Flow Cell Priming kit (ONT, catalog number: EXP-FLP001)
7. Flongle sequencing expansion (ONT, catalog number: EXP-FSE001)
8. NEBNext^®^ quick ligation reaction buffer (New England Biolabs, catalog number: B6058)
9. T4 DNA ligase 2,000,000 units/mL (New England Biolabs, catalog number: M0202)
10. Flongle flow cell (ONT, catalog number: FLO-FLG001)

**Laboratory supplies**
1. Calibrated pipettes single (P1000, P200, P100, P20, P10, P2) (e.g., Rainin)
2. Pipette tips, aerosol resistant, RNase free (P1000, P1000X, P200, P200X, P20) (Rainin, catalog numbers: 30389212, 30389323, 0389239, 30389242, 30389225)
3. 1.5 mL DNA LoBind^®^ tubes (Eppendorf, catalog number: 022431021)
4. Tape Station optical tube strips and tube strip caps (Agilent, catalog number: 401428 and 401425)
5. Tape Station loading tips (Agilent, catalog number: 5067-5153)
6. Qubit tubes (Thermo Fisher, catalog number: Q32856)
7. Nuclease-free water (Thermo Fisher, catalog number: AM9937)
8. Racks for 1.5 mL tubes
9. Magnetic stands (e.g., Thermo Fisher, catalog number: 12321D)
10. Timers (e.g., Cole-Parmer, catalog number: EW-90225-35)
11. Ice or ice packs (e.g., Fisher Scientific, Corning Ice Pan Mini 1L, catalog number: Corning 432116)
12. PPE: Disposable lab coats, nitrile gloves, eye goggles

## Equipment

1. KingFisher^TM^ Duo Prime purification system (Thermo Fisher, catalog number: 5400110)
2. Qubit 4 fluorometer (Thermo Fisher, catalog number: Q33238)
3. Scilogex SCI-M analog microplate mixer (Scilogex, catalog number: 822000049999)
4. Scilogex Mixer accessories: universal circular adapter (Scilogex, catalog number: 18900067)
5. Scilogex Mixer accessories: foam test tube insert for 12 tubes (Scilogex, catalog number: 18900022)
6. Microcentrifuges to hold 1.5–2 mL tubes and 0.2 mL PCR tubes
7. MinION Mk1C (Oxford Nanopore Technologies)
8. GridION (Oxford Nanopore Technologies)
9. Flongle adapter (ONT, catalog number: ADP-FLG001)
10. Cold storage devices: Refrigerator (+4 °C) for flow cells and some reagents, freezer (-20 °C) for enzymes and other reagents, ultra-low freezer (-70 °C) for storage of RNA samples

## Software and datasets

1. MinION Mk1C (or GridION) devices with up to date MinKNOW software
2. The host genome FASTA files were downloaded from GenBank (Table 1), uploaded to MinION Mk1C (or GridION), and then used as references on the adaptive sampling step of the MinKNOW runtime setting to deplete the host sequence reads.
  **Table 1.** Host genome used as references on the adaptive sampling step of MinKNOW

Species	Genome version used
Bovine	Bovine UMD3.1_chromosomes
Swine	GCF_000003025.6_Sscrofa11.1_genomic
Guinea pig	GCF_000151735.1_Cavpor3.0_guineaPig
Sheep	*Ovis aries* (sheep) GCA_016772045.1_ARS-UI_Ramb_v2.0

## Procedure

This protocol was developed with the capacity to rapidly detect and resolve the full genome sequence from an RNA extraction containing full-length FMDV genomes. The whole process takes less than 50 min on the bench, and usually within 25 min to detect the full genome after the run starting on the MinION Mk1C or GridION.

- **Reagent preparation**
  1. Remove the following reagents (Table 2) from the refrigerator and keep at room temperature (RT) for at least 30 min before use.
    **Table 2.** Required reagents stored in a refrigerator

Reagents	Step to use	Note
Qubit RNA HS Assay kit	B.2, F.12	
RNAClean XP beads	C.3, F.1	
NEBNext quick ligation buffer	C.1, D.1, E.1	
Flongle flow cell	G.1	Take one or two extras in case of finding low pores flow cell(s)
  2. Remove the following reagents (Table 3) from the frozen ONT kits and completely thaw before use.
    **Table 3.** Reagents stored in a freezer

Reagents	Step to use	Note
RT adapter (RTA)	C.1	From SQK-RNA002, thaw on ice
RNA adapter mix (RMX)	E.1	From SQK-RNA002, thaw on ice
Wash buffer (WSB)	F.4	From SQK-RNA002, thaw at RT
Elution buffer (ELB)	F.8	From SQK-RNA002, thaw at RT
RNA running buffer (RRB)	G.4	From SQK-RNA002, thaw at RT
Flush tether (FLT)	G.2	From EXP-FLP002, thaw at RT
Flush buffer (FB)	G.2	From EXP-FSE001, thaw at RT
- **Quantification and quality of extracted RNA**
  1. Extract total genomic DNA and RNA from 200 μL of frozen FMDV viral stock for each isolate used (Table 4) using MagMax Core kits following the manufacturer’s instructions on an automated extraction instrument (KingFisher Duo Prime). A final elution of 70 μL of elution buffer provided by the kit is used as the input material for preparation of the libraries.
    **Table 4.** FMDV isolates used in this study

Serotype	Isolate name	Cell line	Cell line origin
A	A 8 Parma	GPVF	Guinea pig
Asia 1	Asia 1, Shamir	BTTP	Bovine
O	O 9 Nueve De Julio	IBRS2	Swine
C	C1 Noville	MVPK	Swine
SAT1	SAT1 Ken 4/98	LK	Sheep
SAT2	Egypt	MVPK	Swine
SAT3	SAT 3 /4 Bech 1/65	IBRS2	Swine
  2. Quantification of the extracted total genomic RNA is performed using the Qubit 4 fluorometer and the Qubit RNA high-sensitivity assay kit, following the manufacturer’s guidelines. The quantity of RNA used in the reaction has been modified from the original ONT protocol; see Note 1 in the General notes and Troubleshooting section below.
- **Primer annealing and ligation**
  1. In a 1.5 mL low-binding tube, add the following reagents and mix by pipetting several times after adding each component.
    NEBNext quick ligation reaction buffer (5×) 3 μL
    RNA 9.5 μL
    RTA 1 μL
    T4 DNA ligase (2M U/mL) 1.5 μL
    Total 15 μL
  2. Put the tube into a microcentrifuge and run the centrifuge for approximately 1 s (briefly spin) to bring down all the components.
  3. Keep the tube at RT for 10 min.
- **Purification with Agencourt RNAClean XP beads**
  1. During the above 10-min incubation time (step C3), prepare the following:
    - Use the 5× NEBNext quick ligation reaction buffer to make 30 μL of 1× ligation buffer by mixing 6 μL of 5× buffer with 24 μL of nuclease-free water.
    - Prepare 70% ethanol from ethyl alcohol by mixing 140 μL of ethanol with 60 μL of nuclease-free water. The wash step requires 150 μL of 70% ethanol per sample.
  2. After the incubation, transfer 25 μL of 1× ligation buffer prepared at step D1a into the ligation tube (step C1) to make a final volume of 40 μL.
  3. Pipette 72 μL of RNAClean XP beads to the above tube for a ratio of 1:1.8; then, tap the tube several times to mix the beads with the sample.
  4. Insert the tube in the test tube foam on a Scilogex mixer and shake at ~300 rpm for 5 min at RT.
  5. Spin down the sample and pellet on a magnetic stand for ~1.5 min. Keep the tube on the magnet and pipette off the supernatant.
  6. Keep the tube on the magnet and wash the beads with 150 μL of 70% ethanol (prepared at step D1b) without disturbing the pellet as described below:
    - Keeping the magnetic stand on the benchtop, rotate the bead-containing tube at 180°. Wait for the beads to migrate toward the magnet and form a pellet.
    - Rotate the tube 180° again (back to the starting position) and wait for the beads to pellet.
  7. Keeping the tube on the magnetic stand, remove the 70% ethanol with a pipette and discard.
  8. Centrifuge briefly, place the tube back on the magnetic stand, and pipette off any residual liquid.
  9. Remove the tube from the magnetic stand and add 23.5 μL of nuclease-free water.
  10. Insert the tube in the test tube foam on a Scilogex mixer and shake it at ~300 rpm for 2 min at RT.
  11. Centrifuge the sample briefly and pellet on a magnetic stand for ~1.5 min.
  12. Transfer 23 μL of the eluate to a new 1.5 mL LoBind tube.
- **Ligation of sequencing adapters**
  1. In the tube with purified RNA (step D12), add the following reagents and mix by pipetting several times after adding each component.
    NEBNext quick ligation reaction buffer (5×) 8 μL
    RMX 6 μL
    T4 DNA ligase (2M U/mL) 3 μL
    Total 40 μL
  2. Put the tube into a microcentrifuge and briefly spin to bring down all the components.
  3. Incubate at RT for 10 min.
- **Purification with Agencourt RNAClean XP beads**
  1. After the incubation (step E3), add 40 μL of RNAClean XP beads to the above tube for a ratio of 1:1; then, tap the tube several times to mix the beads with the sample.
  2. Insert the tube in the test tube foam on a Scilogex mixer and shake at ~300 rpm for 5 min at RT.
  3. Centrifuge the sample and pellet on a magnetic stand for ~1.5 min. Keep the tube on the stand and pipette off the supernatant.
  4. Take the tube from the magnetic stand and add 150 μL of WSB solution. Resuspend the beads completely by flicking the tube, then spin down briefly and put the tube back on the magnetic stand to pellet the beads.
  5. Without disturbing the pellet, discard the supernatant with a pipette.
  6. Repeat steps F4 and F5 once more for a second wash with the WSB solution.
  7. Centrifuge briefly and place the tube back on a magnetic stand; then, remove the residual WSB by pipetting without touching the pellet.
  8. Remove the tube from the magnetic stand and add 17 μL of ELB solution to resuspend the beads.
  9. Insert the tube in the test tube foam on a Scilogex mixer and shake at ~300 rpm for 2 min at RT.
  10. Centrifuge the sample and pellet on a magnetic stand for ~1.5 min.
  11. Transfer the 17 μL of library to a new LoBind tube.
  12. It is optional to measure 1 μL of library in Qubit with the HS RNA kit.
- **Flongle loading**
  1. We changed the MinION/GridION flow cell in the original ONT SQK-RNA002 to Flongle flow cell; see Note 2 in the General Notes and Troubleshooting section below.
  2. Following the ONT manufacturer protocol, put the Flongle flow cell on the MinION Mk1C (or GridION) with a Flongle adapter. Using the MinKNOW software, ensure the flow cell has 50 or more pores and open the sample port.
  3. Prepare the flush buffer by combining 117 μL of FB with 3 μL of FLT. Vortex to mix, then centrifuge briefly.
  4. Use a 200 μL pipette to load 120 μL of mixed flush buffer into the Flongle sample port without introducing any air into the port.
  5. Add an equal volume of RRB buffer to the tube containing the purified library from step F11. Mix well by pipetting and then centrifuge briefly.
  6. Use a 200 μL pipette to load all the library with running buffer into the sample port of the Flongle. Seal the port and start the run.
  7. The runs were performed with the default setting of the MinKNOW software (version: 21.05.12), except the adaptive sampling.
  8. Once the run generates a read file, data analysis can be performed during the runtime. The run can proceed for up to 24 h.

## Data analysis

The base calls were generated using the default setting of the MinKNOW program on the MinION Mk1C or GridION. The data analysis was conducted using the ASFV fast pipeline previously developed by O’Donnell VK et al. with slight modifications and changing the ASFV database to an FMDV isolate database [15]. In short, Minimap2 was used to align reads to sequences of publicly available, complete FMDV genomes obtained from the National Center for Biotechnology Information (NCBI) GenBank as of November 22, 2023. Average alignment score from the resulting alignments was assessed to determine the best reference genome, and data were re-aligned to the corresponding reference genome that had the highest alignment score averaged across the genome length. Average depth of coverage and percent of the genome covered by greater than one read over time were calculated using the default output files from the MinKNOW software and visualized in R.

## Validation of protocol

**Figure 1.**
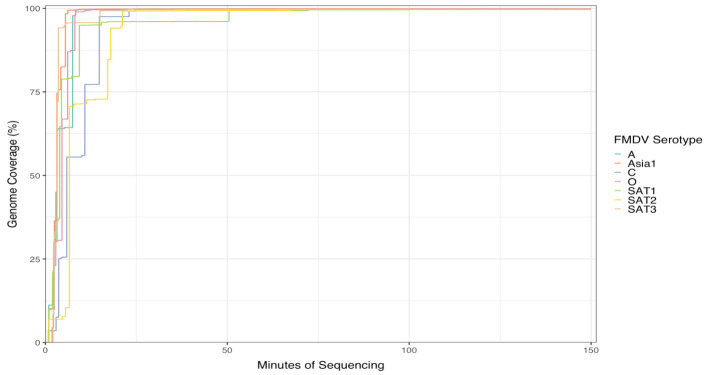
Accumulation of sequencing reads that align to the given foot-and-mouth disease virus (FMDV) reference sequence of the seven serotypes, showing the percentage coverage of the reference genomes.

**Figure 2.**
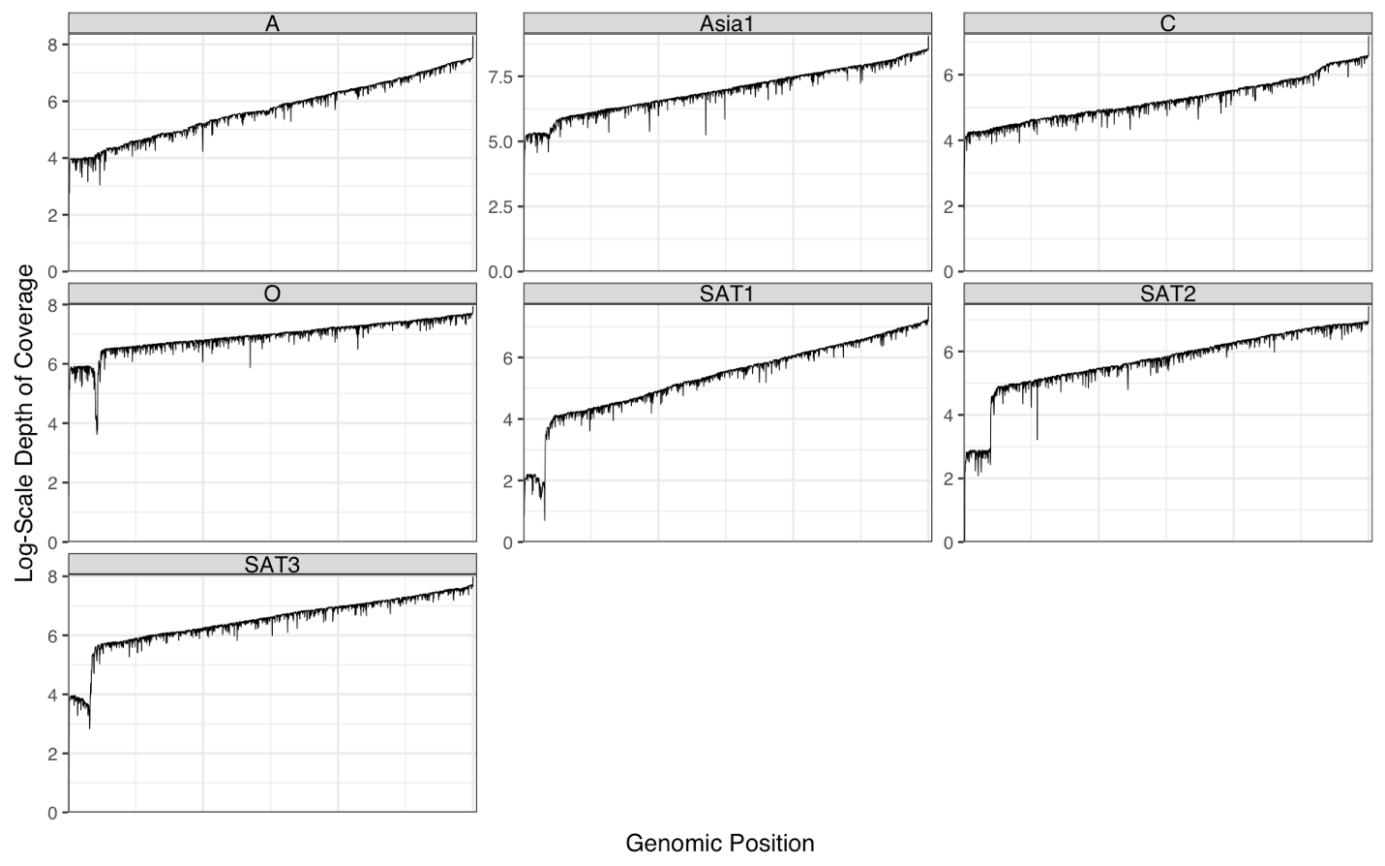
Coverage depth on the natural log scale for the reference genome sequence of the seven Flongle runs. Due to the different size of the reference isolates used (see Table 5), the x-axis is normalized to the percentage of the given isolate genome: starting from 0% and ending at 100% of the genome sequence length.

The seven FMDV isolates used in this study are listed in Table 5 with the condition of the seven Flongle runs performed with the protocol above on fresh RNA extractions from seven FMDV serotypes. The analyzed run results are displayed in Figures 1 and 2. All serotype genomes were resolved within 25 min of runtime, and the data generated were enough to cover the full genome of the reference strain used, except the SAT 1 sample that had over 90% genome coverage after 25 min and reached 99.8 % coverage after 50 min (Figure 1). The reads coverage depth of the reference genomes are displayed in Figure 2: one graph for each serotype. There was increased coverage on the 3′ end of the genome vs. the 5′ end, since ONT RNA Direct Sequencing kit requires the RNA molecule to have a poly-A tail at the 3′ end, and any FMDV RNA splitting forms without poly-A could not be sequenced. To better display the depth of coverage, the y-axis of Figure 2 is displayed on the natural log scale, with unit 0 representing 1 read coverage and unit 8 representing ~2981 read coverage. Although only FMDV isolates were used in the study, this method may be used to sequence any viral RNA with a poly-A tail.

**Table 5.** Summary of the seven FMDV samples direct RNA sequencing runs. The adaptive sampling option was used in the MinKNOW software of MinION or GridION to deplete the reads that align to the host genome—the cell line used to generate the viral stocks. The last two columns display the analysis results of the fastq files of each sample after running for 24 h and passing the ONT default quality controls, aligned to the given reference sequence.

FMDV serotype	Input RNA (ng)	Library loaded (ng)	Adaptive sampling	Reference genome	Average depth	Percentage of genome coverage to reference
A	176	86.4	Guinea pig	AY593792	1553	99.9
Asia 1	458	186	Bovine	JF739177	3499	99.9
O	500	150	Swine	AY593819	1573	99.9
C	293	93.3	Swine	AY593804	463	99.8
SAT1	327	122	Sheep	KM268899	975	99.8
SAT2	274	165	Swine	JX014255	1453	99.8
SAT3	125	63	Swine	AY593853	953	99.8

## General notes and troubleshooting

1. The ONT SQK-RNA002 original protocol recommends 500 ng of poly-A+ tailed RNA in 9 μL for library preparation. We used 9.5 μL of RNA by omitting the control RNA provided in the kit, and as low as 125 ng total RNA for the library preparation.
2. We ran the RNA library (Step G in the Procedure above) on a Flongle flow cell instead of a MinION/GridION flow cell. As a result, the cost of flow cell alone for our protocol was reduced dramatically, as a Flongle costs ~1/10 of a MinION/GridION flow cell. In addition, during our tests, as low as 63 ng of library was loaded into the Flongle (see Table 5 below), compared to 200 ng in the original protocol, which generated enough sequencing data to cover the full length of the FMDV genome (Table 5, Figures 1 and 2).
**General notes**
  Initially, the adaptive sampling option was enabled on MinKNOW to deplete the reads aligned to the host (cell line origin) genome after uploading the host fasta files to the MinION Mk1C or GridION, but this step was determined to be optional since the adaptive sampling has no significant impact on the final result (data not shown).
  The quality and quantity of FMDV genomic RNA are critical for RNA direct sequencing. Real-time reverse transcription PCR (RT-qPCR) [16] is commonly used to detect FMDV and assess RNA quality and quantity. However, there is no direct correlation between NGS (including RNA direct sequencing) results and Ct values. RT-qPCR relies on specific primers/probes to amplify a short sequence (usually around 100 bp), while RNA direct sequencing is independent of the target sequence. Mutations in the primer/probe site can cause RT-qPCR failure or abnormally high Ct values, but NGS can still achieve overall genome sequencing. In our experience, samples with low Ct values sometimes yield poor NGS data due to viral genome degradation, even when the short region targeted by RT-qPCR primers/probe remains amplifiable. Ultimately, RNA direct sequencing data quality depends on factors beyond Ct values.
**Troubleshooting**

- When encountering low bead recovery during the XP beads purification in the Primer annealing step (step D), consider the following two factors to ensure optimal results:
  1) Homogenization of stock beads solution before adding it to the reaction. When the AMPure beads-to-sample ratio falls below 0.4:1, there will be no nucleic acid recovered by the beads at all.
  2) At least 70% ethanol concentration in the washing solution. If the ethanol concentration is lower, nucleic acid may be eluted from the beads during washing. The reduced ethanol concentration in washing buffer could be caused by several factors, such as an older solution not freshly made during the same day of the experiment; the stock of ethanol used to prepare the washing solution has a lower concentration than indicated on the label due to prolonged storage or inadequate sealing after previous usages.
